# Domestication of medicinal plants (*Lonicera japonica* Thunb.) in China: comparison of morphological, resistance and biochemical traits between wild and cultivated populations

**DOI:** 10.3389/fpls.2024.1501396

**Published:** 2025-01-24

**Authors:** Congzhe Hou, Luyao Huang, Zhuangzhuang Li, Nan Sun, Sheng Yang, Jia Li, Zhenhua Liu

**Affiliations:** ^1^ Experimental Center of Shandong University of Traditional Chinese Medicine, Jinan, China; ^2^ School of Pharmacy, Shandong University of Traditional Chinese Medicine, Jinan, China

**Keywords:** honeysuckle, wild, cultivated, domestication, biochemical composition

## Abstract

**Background:**

Most studies on domesticated plants have focused on those utilized for sustenance purposes. This study provides valuable insights into the domestication processes of honeysuckle (*Lonicera japonica* Thunb.), a semi-evergreen twining vine in the Caprifoliaceae family that is important in traditional Chinese medicine for its flower buds and potential as a dietary supplement. The definition of domestication syndrome for honeysuckle remains unclear due to its perennial and asexual reproduction mode, resulting in a lack of information on domestication-related traits. Therefore, this study aims to compare and analyze differences in morphology, resistance, and biochemical composition between wild and cultivated varieties.

**Methods:**

A total of 36 wild and 81 cultivated specimens were examined to compare plant morphology, flowering time, bud length, active ingredients, and resistance between wild and cultivated populations.

**Results:**

The cultivated honeysuckle exhibited several noteworthy characteristics, including more erect plant morphology, higher flower-to-leaf ratio, more obvious aggregation of flowers into clusters at the top of branches, higher frequency of flowering, and longer bud length compared to the wild type. Additionally, the cultivated specimen demonstrated significantly elevated levels of chlorogenic acid as a biochemical constituent. However, in terms of resistance against powdery mildew, wild individuals displayed evident advantages over their cultivated counterparts.

**Conclusion:**

The observed phenotypic variation within the honeysuckle field provides empirical support for the hypothesis that farmer management practices influence domestication syndrome, as indicated by the deliberate enhancement of specific desirable traits during species domestication.

## 1 Introduction

Plant domestication is a protracted evolutionary process aimed at fulfilling human preferences and needs, entailing the transformation of wild individuals into cultivated ones through continuous selection for desired traits. Domestication can be perceived as an ongoing progression that commences with the preservation and nurturing of a species within its natural habitat (Siqueira et al., 2023). The transition from wild to cultivated state entails numerous morphological and genetic alterations, commonly referred to as domestication syndrome (Meyer et al., 2012). Cultivating using wild population material in a human environment involves successive generations being selectively bred for traits deemed valuable by humans (Abbo et al., 2014). Throughout this process, there exists a continual and progressively widening disparity between wild and domesticated populations, spanning from initial domestication to complete acclimatization. Presently, the evolutionary and genetic aspects of domestication processes have been extensively investigated in temperate cereal crops and legumes, rendering these crops well comprehended (Vaughan et al., 2007; Meyer et al., 2012; Muñoz et al., 2017). In contrast, our understanding of the domestication process for perennial crops remains limited, despite their significant contribution to global human nutrition.

Honeysuckle, perennial semi-evergreen twine vine, belongs to the caprifoliaceae family and is widely utilized in traditional Chinese medicine for its dried bud or flower with initial blooming. These botanical materials are extensively employed in clinical applications due to their remarkable properties of heat-clearing, detoxification, blood-cooling, and anti-inflammatory effects (Choi et al., 2010; Li et al., 2020; Kim et al., 2022; Yang et al., 2023). The earliest record of artificial cultivation can be found in the Song Dynasty’s “Su Shen Neihan Liangfang”, specifically in volume 9 which states: “The roots can be transplanted to the court as a preparation for emergencies.” Subsequently, accounts of large-scale planting of honeysuckle are documented in local county annals. For instance, according to the “County Annals of Fei County” (formerly known as Pingyi and Fei County), there is an entry from Guangxu year 22 that mentions: “The name “honeysuckle” is derived from the fact that the flower undergoes a color transformation, starting off as white and eventually turning yellow. In the past, it was also used for tea due to its abundance. During the reign of Jiaqing, business travelers frequently achieved substantial profits through trading it elsewhere, thereby resulting in numerous instances of multiple cultivation locations within a short span of time. This history spans over two centuries in Fei County located in Shandong Province.

Honeysuckle has been collected for medicinal purposes, and through a gradual process of selecting wild individuals with intriguing characteristics for protection and cultivation, the domestication of this plant began. Compared to wild honeysuckle, cultivated typically exhibit reduced levels of genetic variation, primarily attributed to the “domestication bottleneck” phenomenon resulting from the introduction of a limited number of wild individuals possessing desirable traits. Conversely, cultivation generally enhances user preference traits (e.g., flower bud size) compared to wild forms. Consequently, cultivated honeysuckle flower buds tend to be larger in size and possess higher concentrations of bioactive constituents. The presence of phenotypic variation in the field provides support for the hypothesis that domestication syndrome is driven by farmer management practices and manifested through the prevalence of specific traits favored during species intensification. The evolutionary history of domestication can be explored through a comparison of morphological and genetic differences between cultivated strains and their original wild honeysuckle, revealing that these disparities may arise from human selection on cultivated honeysuckle and the influence of selection pressure imposed by new environmental conditions. To test this hypothesis, we conducted a comprehensive comparative study on wild and cultivated honeysuckle plants, encompassing plant type, flower bud size, flower distribution, main functional components, as well as stress resistance – all traits specifically selected during the domestication of honeysuckle. We assess the distinct characteristics of domesticated populations using this traditional methodology, examining whether human selection has enhanced the phenotypic variability of selected traits within these populations.

## 2 Materials and methods

### 2.1 Source of materials

**Figure 1:**
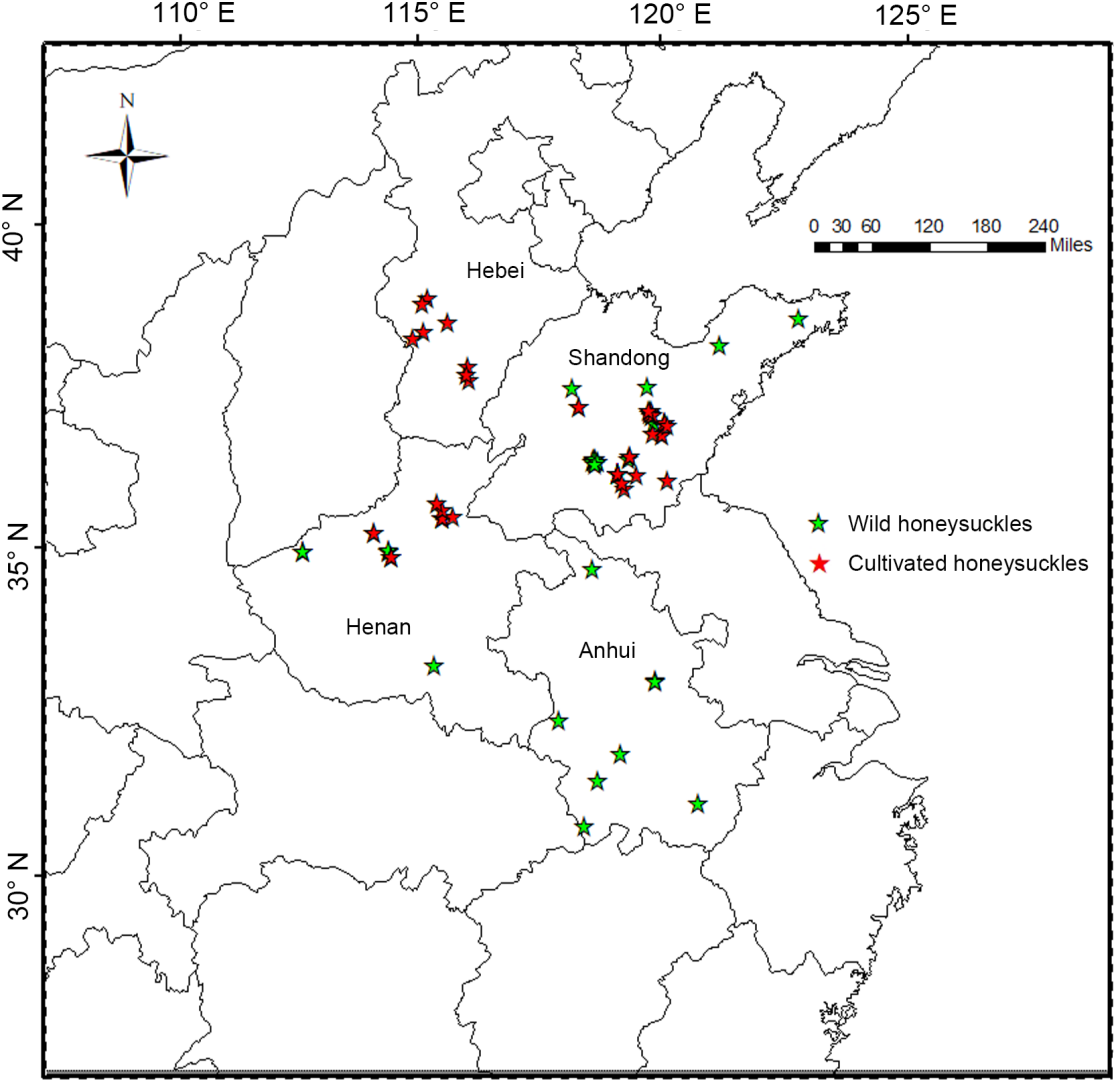
Sampling sites in Hebei, Shandong, Henan and Anhui province of China.

In this study, samples of wild and cultivated honeysuckle from diverse locations were random selected (**Figure 1**). A wild individual is classified when found in a location without any signs of human habitation or evidence of past or present specimen management by local residents. Conversely, cultivated individuals in agroforestry systems are plants managed by locals. A total of 117 honeysuckle specimens were collected, including 81 cultivated and 36 wild. The geographical coordinates were recorded for each sample, and the collected honeysuckle branches were propagated from cuttings at the honeysuckle germplasm resource bank of Shandong University of Traditional Chinese Medicine. The statistical analysis of biological characteristics was conducted two years after the branches planted.

### 2.2 Horticultural character statistics of honeysuckle

*Lonicerae Japonicae* Flos., known as Jinyinhua in the Chinese Pharmacopeia, refers to the dried flower buds or the initial flowers of *Lonicera japonica* Thunb. Key horticultural traits in production include:plant type and flower clustering status along the branches, which are associated with collecting efficiency; the ratio of flower to leaf numbers, the frequency of flowering per year and the honeysuckle bud length, which are associated with the yield.

Plant type: The plant type of honeysuckle was divided into upright type and creeping type, which are distinguished easily, and could be directly observed.

The ratio of flower to leaf numbers in May is an important index of honeysuckle yield, which can be divided into three types: greater than 50% is defined as high, 30-50% is defined as medium, and less than 30% is low.

Flower clustering status along the branches is important for the flower picking efficiency. According to the percentage of clustered flower branches (**Supplementary Figure S1**), honeysuckle can be categorized into three levels: greater than 50% is defined as strong clustered, 20-50% is defined as medium clustered, and less than 20% is weak clustered.

The frequency of flowering per year: the annual flowering times of cultivated and wild honeysuckle were counted and recorded.

The honeysuckle bud length is closely related to the weight of individual flowers, and can be easily and accurately measured, thus considered as a major index in production. The length of honeysuckle flower buds (at the pre-blooming white stage) was quantified using ImageJ software for statistical analysis, with at least three repeated measurements and subsequent averaging.

### 2.3 Powdery mildew infection index statistics

The statistics on powdery mildew infection were based on two years’ natural infection in the field. Classification of powdery mildew disease in honeysuckle: Grade 0 indicates absence of disease; Grade 1 represents lesion area accounting for less than 20% of the entire plant; Grade 2 signifies lesion area accounting for 20%-40% of the entire plant; Grade 3 denotes lesion area accounting for 40%-60% of the entire plant; Grade 4 represents lesion area exceed more than 60% of the entire plant.

### 2.4 Detecting the main phytochemical components of honeysuckle

The flowers at the same developmental stage (pre-blooming white stage) were collected from each specimen simultaneously in May and subsequently dried. Contents of primary pharmaceutical components were established using an Agilent 1200 HPLC system (Agilent Technologies, Santa Clara, CA), fitted with the Agilent 1260 Infinity II Quadrupole Pump and DAD Detector with a diode-array detector following the protocol by Guo et al., 2022 (Guo et al., 2022). The primary phytochemical components of honeysuckle were shown in **Table 1**.

**Table 1:** The primary phytochemical components of honeysuckle.

Common name	Specific name	Chemical formula
Chlorogenic acid	3-O-Caffeoylquinic acid	C_16_H_18_O_9_
Protocatechuic acid	Protocatechuic acid	C_7_H_6_O_4_
Isochlorogenic acid A	3,5-Di-O-caffeoylquinic acid	C_25_H_24_O_12_
Isochlorogenic acid B	3,4-Di-O-caffeoylquinic acid	C_25_H_24_O_12_
Isochlorogenic acid C	4,5-Di-O-caffeoylquinic acid	C_25_H_24_O_12_
Luteoloside	Luteolin 7-glucoside	C_21_H_20_O_11_
Rutin	Rutin	C_27_H_30_O_16_
Lonicerin	Luteolin 7-O-rutinoside	C_27_H_30_O_15_
Secologanic acid	Secologanoside 7-methyl ester	C_16_H_22_O_11_
Sweroside	Sweroside	C_16_H_22_O_9_
Secoxyloganin	Secoxyloganin	C_17_H_24_O_11_

### 2.5 Statistical analysis

The statistical analyses were performed using SPSS software (Version 25.0, SPSS, Chicago, IL, USA) on a Windows operating system. Student’s t-test was employed to analyze the data simulated from a normal distribution, while the nonparametric Mann-Whitney U test was used for data that did not meet the assumption of normality. The Chi-square test was utilized to compare frequencies between groups. Results are presented as mean ± standard deviation or number (percentage). Statistical significance was determined based on two-sided p-values<0.05. Principal component analysis (PCA) and multiple correspondence analysis (MCA) were performed using R software (R 4.1.1).

## 3 Results

### 3.1 Comparative analysis of botanical characteristics

The findings indicate significant morphological differences between wild and cultivated honeysuckle. Wild individuals typically exhibit a creeping growth habit, whereas cultivated individuals predominantly display an upright form, with flowers often clustered at the top of branches, with a high flower-to-leaf ratio (**Table 2**). The annual flowering frequency of the cultivated honeysuckles surpasses that of wild counterpart significantly (**Table 3**).

**Table 2:** Morphometric characteristics in wild and cultivated populations of honeysuckle. .

	Wild			Cultivated			*χ^2^*	*P*
Plant type	Erect type		Creeping type	Erect type		Creeping type	94.904^a^	<0.001
	5.56% (2/36)		94.44% (34/36)	96.30% (78/81)		3.70% (3/81)		
Ratio of flower to leaves	High	Medium	Low	High	Medium	Low	20.987^a^	<0.001
	22.22% (8/36)	41.67% (15/36)	36.11% (13/36)	61.73% (50/81)	25.93% (21/81)	12.35% (10/81)
Flower of apical clustering	Strong	Medium	Weak	Strong	Medium	Weak	19.025^a^	<0.001
	5.56% (2/36)	52.78% (19/36)	41.67% (15/36)	41.98% (34/81)	43.21% (35/81)	14.81% (12/81)

**Table 3:** Comparison of flowering frequency in wild and cultivated populations of honeysuckle.

	Flowering times per year					*χ^2^*	*P*
	1	2	3	4	5		
Wild	19.44% (7/36)	75% (27/36)	5.56% (2/36)	0% (0/36)	0% (0/36)	48.477^a^	<0.001
Cultivated	1.23% (1/81)	25.93% (21/81)	51.85% (42/81)	18.52% (15/81)	2.47% (2/81)		

### 3.2 Comparison of powdery mildew infection traits

The incidence of powdery mildew in honeysuckle was determined in this study. The results revealed that the cultivated individuals exhibited an incidence rate of 71.05%, whereas the wild had a lower rate of 33.33%. Furthermore, as depicted in **Table 4**, the ratio of severely infected plants among cultivate was significantly higher compared to that among wild. These findings indicate a notable disparity between the resistance levels of cultivated and wild individuals towards powdery mildew.

**Table 4:** The comparison of powdery mildew infection traits in wild and cultivated honeysuckle individuals.

Group	Diseaze Index					Incidence rate	*χ^2^*	*P*
	0	1	2	3	4			
Wild	24	11	1	0	0	33.33%	23.783a	<0.001
Cultivated	22	23	15	1	20	71.05%

**Figure 2:**
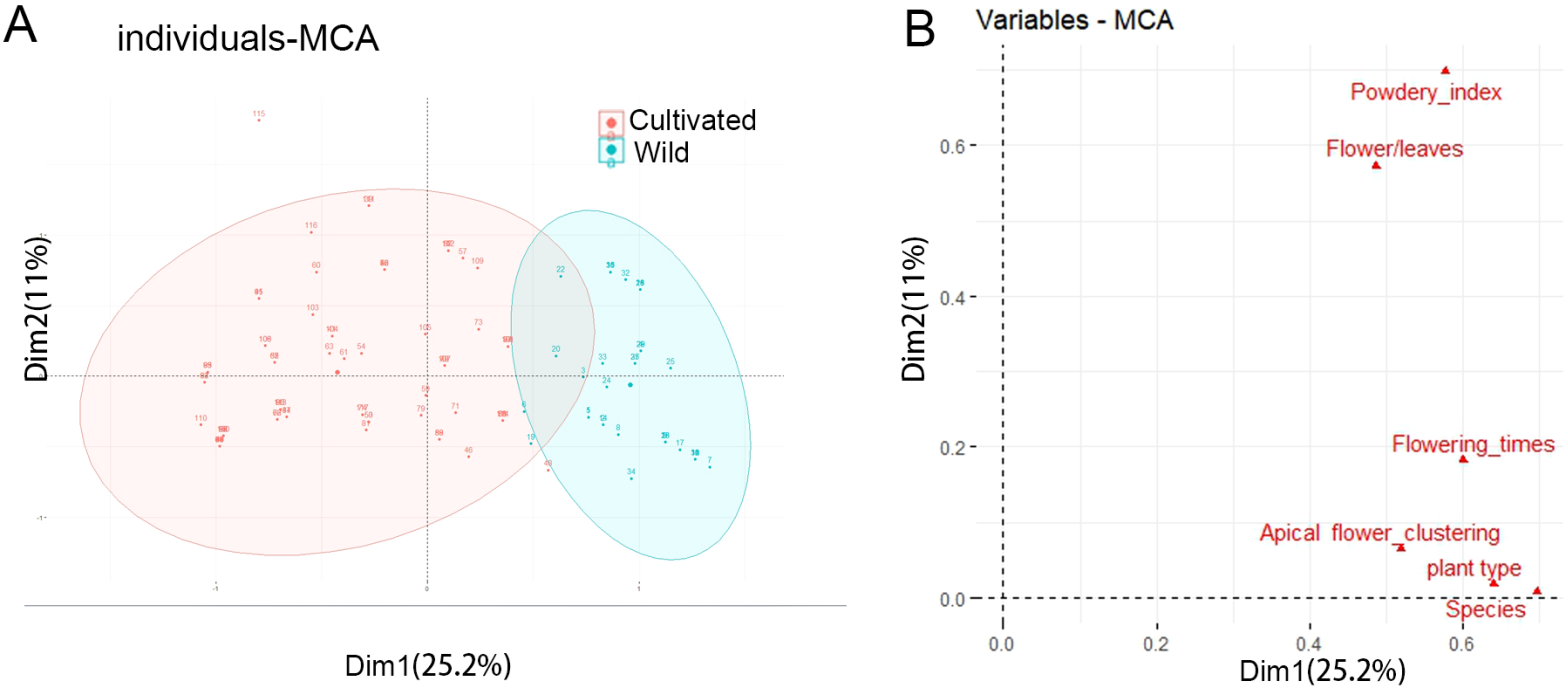
Classification of wild and cultivated honeysuckles by MCA analyses. **(A)** Discrimination of wild and cultivated honeysuckle from the MCA according to axes 1 and 2. **(B)** Correlation between variables and the dimensions.

The MCA (Multiple Correspondence Analysis) explains 36.2% of the total variance, with25.2% for axis 1 and 11% for axis 2 (**Figure 2A**). Axis 1 differentiating wild and cultivated individuals (**Figure 2A**). The variables Plant type and Flowering times are the most correlated with dimension 1. Similarly, the variables Powdery index and Flower/Leaves are the most correlated with dimension 2 (**Figure 2B**).

### 3.3 Comparison of flower bud length in white stage of honeysuckle

**Figure 3:**
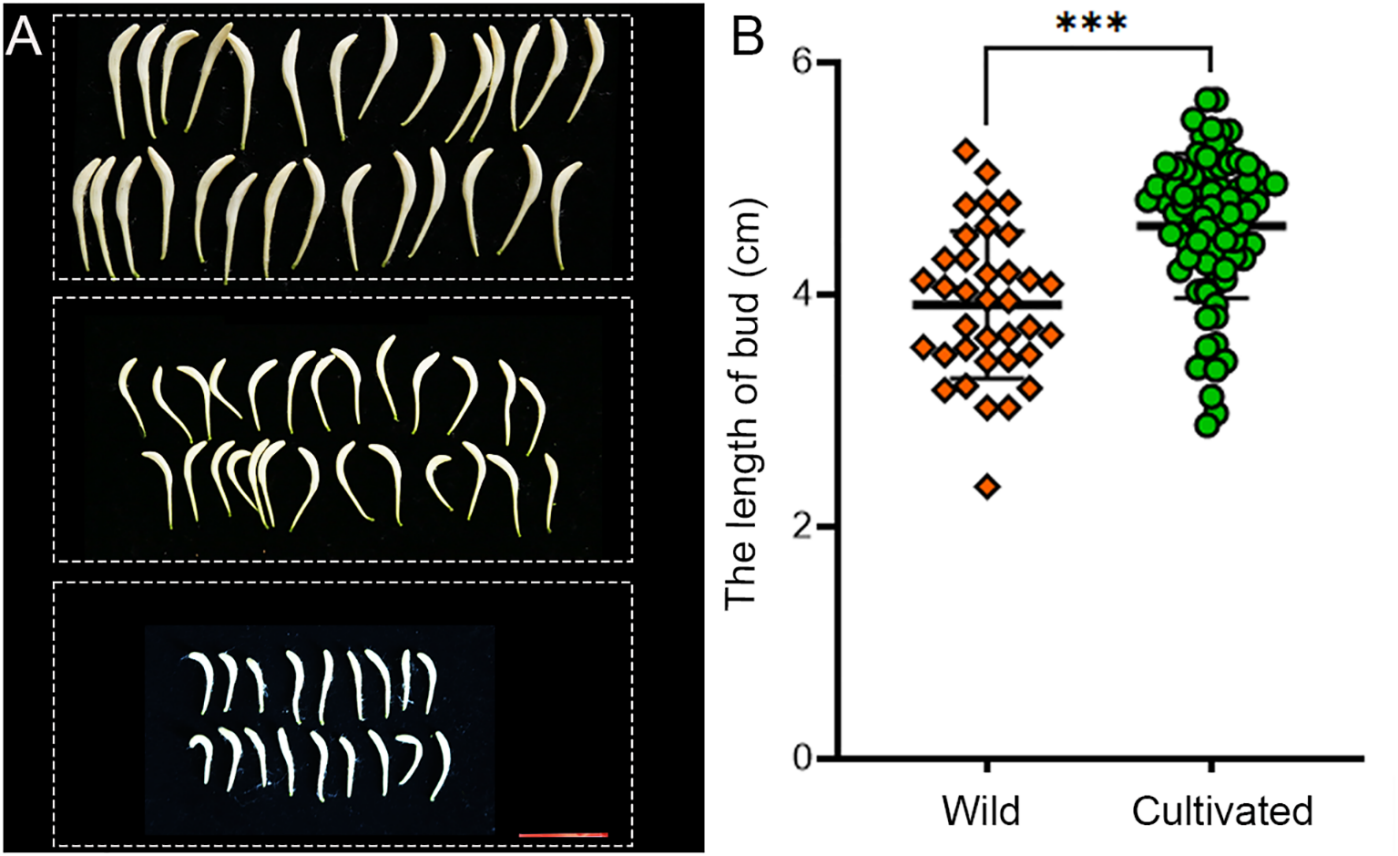
Comparison of bud length between wild and cultivated honeysuckles. **(A)** The honeysuckle buds were obtained from three distinct specimens, each set displaying varying lengths. scale bar 2.5 cm. **(B)** Statistical results revealed a significant disparity in the length of honeysuckle buds between wild (n=36) and cultivated (n=81) specimens. ^***^ Student’s *t* test, *P* < 0.001.

The statistical analysis reveals that the average length of flower buds in wild populations is 3.794 ± 0.628 cm, compared to 4.537 ± 0.641 cm in cultivated populations (**Figure 3**). Wild honeysuckles exhibit significantly shorter flower buds compared to their cultivated counterparts.

### 3.4 Comparison of phytochemical components in honeysuckle

**Figure 4:**
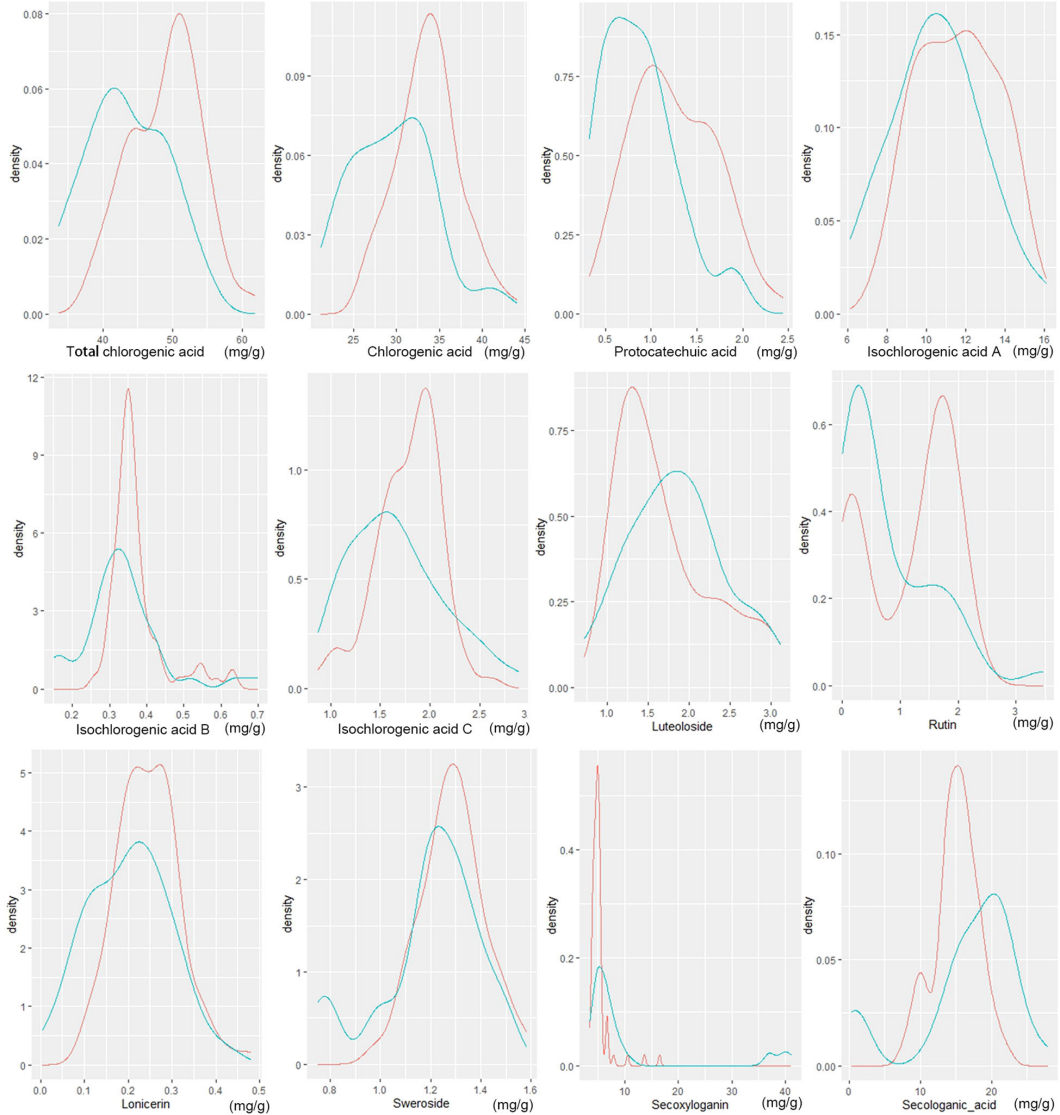
Distribution of wild (green line) and cultivated (red line) honeysuckles with different component content. The horizontal axis represents the content of the component, while the vertical axis indicates the density of the data. Density refers to the probability that data points are distributed within a specific interval around a certain value.

Honeysuckle contains a variety of bioactive compounds, primarily including phenolic acids, flavonoids, and iridoid glycosides. In this study, the three principal chemical constituents were identified and quantified in flower bud samples from all honeysuckle germplasm (**Figure 4**). Notably, significant differences were observed in the content of these key chemical components between wild and cultivated honeysuckle.

The total content of chlorogenic acid in the flower buds of wild and cultivated honeysuckle was quantified at 43.45 ± 5.69 mg/g and 48.98 ± 5.09 mg/g, respectively. The levels of chlorogenic acid were determined to be 29.52 ± 4.80 mg/g and 33.70 ± 3.72 mg/g, respectively, while the protocatechuic acid content were measured at 0.87 ± 0.40 mg/g and 1.25 ± 0.46 mg/g, respectively. The contents of isochlorogenic acid A were found to be at levels of approximately 10.54 ± 2.32 mg/g for wild honeysuckle and 11.60 ± 2.06 mg/g for cultivated honeysuckle, whereas the contents of isochlorogenic acid B were approximately 0.34 ± 0.11 mg/g for wild honeysuckle and 0.37 ± 0.073 mg/g for cultivated honeysuckle. The concentrations of the aforementioned five of phenolic acids in wild individuals were lower than that in cultivated individuals, and there was a significant difference (*P*<0.05), accompanied by a wider range of variation. However, no significant differences were noted in the contents of isochlorogenic acids C (**Table 5**).

**Table 5:** Comparison of phytochemical components in wild (n=36) and cultivated (n=81) honeysuckles.

Compounds (mg/g)	Mean±SD (mg/g)		Coefficient of variation		
	Wild	Cultivated	Wild	Cultivated	*P*
Total Chlorogenic acid	43.45 ± 5.69	48.98 ± 5.09	13.09%	10.40%	<0.0001
Chlorogenic acid	29.52 ± 4.80	33.70 ± 3.72	16.25%	11.03%	<0.0001
Protocatechuic acid	0.87 ± 0.40	1.25 ± 0.46	46.04%	36.64%	<0.0001
Isochlorogenic acid A	10.54±2.32	11.60±2.06	21.97%	17.77%	0.0147
Isochlorogenic acid B	0.34±0.11	0.37±0.073	33.30%	19.56%	0.0093
Isochlorogenic acid C	1.66±0.47	1.78±0.32	28.41%	17.86%	0.1129
Luteoloside	1.84±0.59	1.70±0.58	32.13%	34.11%	0.1128
Rutin	0.78±0.80	1.18±0.758	102.90%	64%	0.0308
Lonicerin	0.20±0.09	0.25±0.075	46.43%	29.15%	0.0045
Sweroside	1.20±0.20	1.29±0.13	16.55%	9.86%	0.0061
Secoxyloganin	10.65±11.62	5.16±1.886	109.10%	36.49%	<0.0001
Secologanic acid	16.58±7.24	15.10±3.06	43.69%	20.25%	0.0015

Each sample in each group was detected in triplicate.

Luteoloside, rutin, and lonicerin are the predominant flavonoids compounds found in honeysuckle. The concentration of luteoloside in the flower buds of wild and cultivated honeysuckle was measured at 1.84 ± 0.59 mg/g and 1.70 ± 0.58 mg/g, while no statistically significant difference was observed in luteoloside levels between the two categories (**Table 5**). The rutin concentration in the flower buds of wild and cultivated honeysuckle was measured as 0.78 ± 0.80 mg/g and 1.18 ± 0.758 mg/g, respectively, while the lonicerin content was recorded as 0.20 ± 0.093 mg/g and 0.25 ± 0.075 mg/g, respectively. The two components exhibit a statistically significant yet limited disparity between wild and cultivated varieties, with a lower coefficient of variation observed in cultivated varieties compared to their wild counterparts (**Table 5**).

The iridoid glycosides present in honeysuckle include sweroside, secoxyloganin, and secologanic acid. The concentration of sweroside in the flower buds of wild and cultivated honeysuckle was determined to be 1.20 ± 0.20 mg/g and 1.29 ± 0.13 mg/g, respectively. The secoxyloganin concentration in the flower buds of wild and cultivated honeysuckle was measured as 10.65 ± 11.62 mg/g and 5.16 ± 1.88 mg/g, while the secologanic acid content was recorded as 16.58 ± 7.24 mg/g and 15.10 ± 3.06 mg/g. The three exhibit notable differences, with the coefficient of variation being greater in wild honeysuckle (**Table 5**).

**Figure 5:**
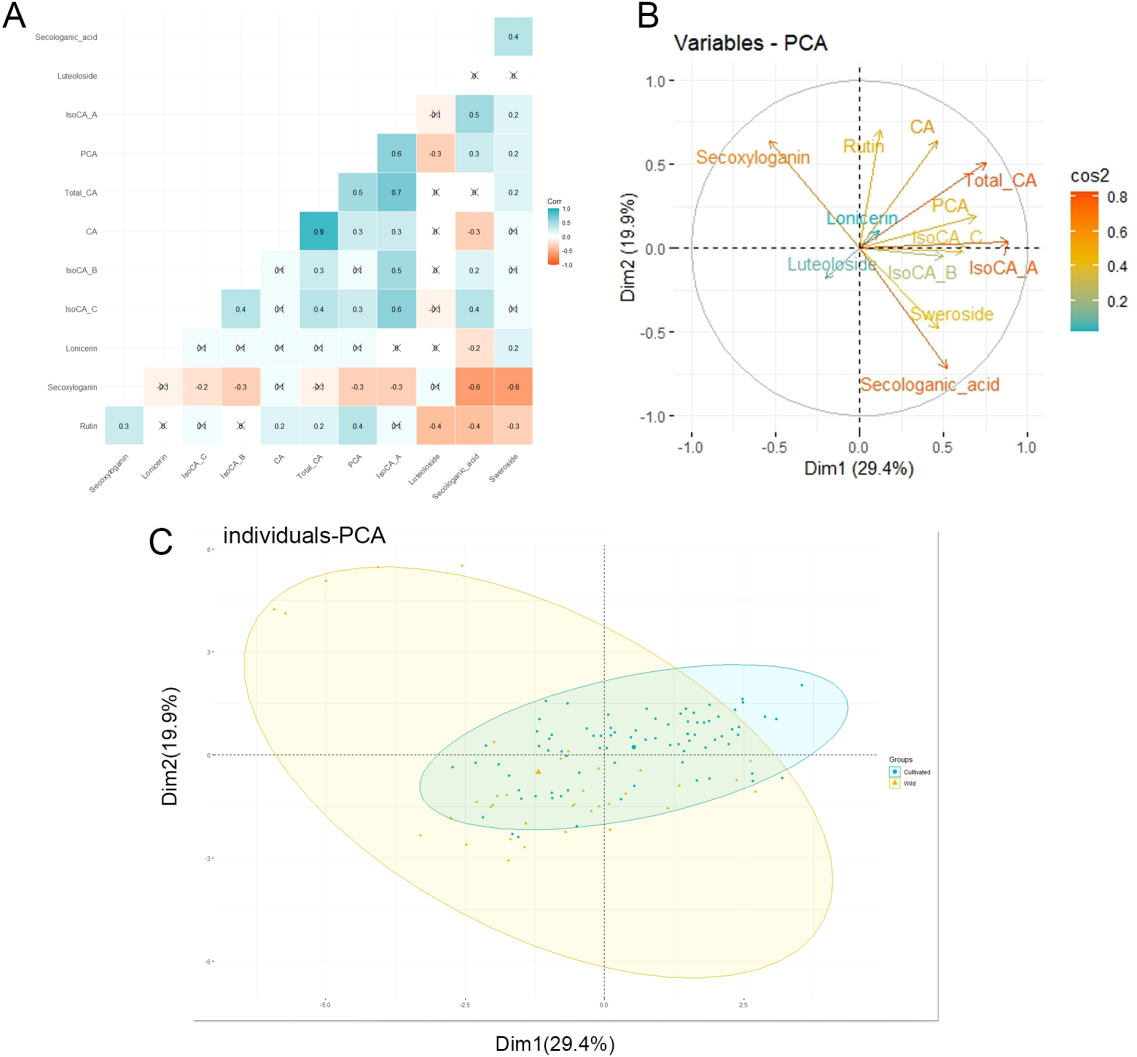
Correlation analysis and principal component analysis. **(A)** The heat map shows the correlation analysis between the components. **(B)** Correlation and contribution of variables to the formation of axes 1 and 2. **(C)** PCA (Principal component analysis) separated individuals according to individual components content. Total_CA, Total chlorogenic acid; CA, Chlorogenic acid; PCA, Protocatechuic acid; IsoCA_A, Isochlorogenic acid A; IsoCA_B, Isochlorogenic acid B; IsoCA_C, Isochlorogenic acid C.

The correlation of the components obtained in this study was analyzed, and the results are displayed as a correlation heat map (**Figure 5A**). The individuals were segregated based on the content of their components in Principal Component Analysis. The first two components account for approximately 49.31% of the total variance (29.41% for axis 1 and 19.9% for axis 2) (**Figure 5B**). However, the PCA did not differentiate between wild and cultivated individuals based on their component content (**Figure 5C**).

## 4 Discussion

Honeysuckle was initially documented in the “Famous doctor records” where Hongjing Tao described it as a plant that remains evergreen even during winter. In the early stages, wild honeysuckle resources were abundant, and collection primarily focused on stems and leaves for medicinal purposes. Large-scale cultivation of honeysuckle began in Fei County in Shandong Province around 1796, marking a history spanning over 200 years. Consequently, the domestication history of honeysuckle is comparatively shorter than that of other cultivated perennial cash crops.

### 4.1 Effect of selection on morphological parameters of honeysuckle

In the horticultural character of honeysuckle, we observed clear indications of domestication syndrome: the cultivated honeysuckle exhibited a more erect growth habit, a higher ratio of flowers to leaves, increased frequency of flowering throughout the year, clusters of flowers formed top of branches, and significantly longer flower bud length compared to wild populations. This shift in morphology can be interpreted as a manifestation of domestication syndrome resulting from long-term cultivation, selective breeding, and human-mediated dispersal. Previous studies have demonstrated that during the cultivation process, ease of harvesting is often an important criterion for human selection in honeysuckle plants. In general, cultivars exhibiting an erect growth habit, a high ratio of flowers to branches, and clustered inflorescences are more advantageous for harvesting purposes, thereby exemplifying the domestication syndrome.

The results of our study demonstrate a significantly higher frequency of flowering in cultivated honeysuckle compared to their wild counterparts, which can be attributed to the process of domestication. The challenging living conditions faced by wild honeysuckle necessitate the accumulation of sufficient nutrients for survival and resistance against natural or biological factors. In contrast, cultivated specimen enjoy a more favorable living environment with human intervention ensuring their survival, resulting in a greater number of annual flowering periods compared to the wild type. This not only enhances honeysuckle production but also provides doctors with increased opportunities for patient treatment during an era when storage technology was underdeveloped.

Furthermore, the increased length of flower buds in cultivated honeysuckles compared to wild type aligns with expectations for larger edible fruits in trees. This phenomenon can be attributed to the domestication syndrome induced by historical cultivation practices and human selection (Parker et al., 2010; Pedrosa et al., 2018; Mboujda et al., 2022). One possible factor contributing to the increase in bud organ size in cultivated populations could be reduced levels of environmental stress (Purugganan and Fuller, 2009), thereby allowing for greater allocation of resources towards flowering. Furthermore, honeysuckle bud size is a key trait of interest for both farmers and consumers. The findings presented here support the hypothesis that this observed variation may be attributed to human practices, which typically favor the selection of individuals with easily harvestable and larger flower buds (Abbo et al., 2014). Such variation based on medicinally valuable parts is commonly observed in most perennial cash crops. These differences may be associated with cultivation management practices such as fertilization and irrigation, as well as variations in genetic backgrounds.

### 4.2 Effect of selection on the phytochemical components of honeysuckle

The ancients revered honeysuckle as a pivotal herb for treating plague, possessing the ability to dispel wind heat, clear heat and detoxify the body. It was particularly suitable for managing febrile diseases such as fever and cold. Honeysuckle contains various active components including phenolic acids, flavonoids, volatile oils, and triterpenoid saponins. Phenolic acids are significant secondary metabolites in honeysuckle that exhibit remarkable anti-inflammatory activity due to their structural composition containing multiple phenolic hydroxyl groups (Guo et al., 2015; Li et al., 2023). Among these compounds, chlorogenic acid is the principal effective component which often determines the quality and efficacy of medicinal materials. According to the Chinese Pharmacopoeia (2020) guidelines, honeysuckle should contain no less than 1.5% chlorogenic acid (C16H18O9) content and a total chlorogenic acid (chlorogenic acid (C16H18O9), isochlorogenic acid A, isochlorogenic acid C) content not less than 3.8%. The present study demonstrates that cultivated individuals exhibit higher levels of chlorogenic acid and total chlorogenic acid compared to wild individuals, indicating a distinct composition resulting from domestication syndrome. Through long-term cultivation and selection, humans have often derived efficient germplasm from wild sources and developed them into cultivated varieties, thereby subtly influencing the selection of effective components in cultivated flower buds. Additionally, this indirectly substantiates the significance of chlorogenic acid as a pivotal bioactive compound in honeysuckle’s therapeutic properties. Early physicians lacked sophisticated equipment to quantify the levels of active ingredients in honeysuckle; Therefore, their assessment of its efficacy was solely based on therapeutic effects, while the selection of honeysuckle plants with high effective components was also done indirectly. Consequently, artificially cultivating honeysuckle through cuttage or transplanting emerged as an initial step towards domestication.

### 4.3 Effect of selection on resistance of honeysuckle

Honeysuckle exhibits remarkable environmental adaptability, demonstrating exceptional cold tolerance, drought resistance, salt and alkali tolerance. Its inherent advantage lies in its ability to acclimate to various climates. The focus of this study is on powdery mildew susceptibility. Our findings reveal that the cultivated honeysuckle population has experienced a loss of stress-related genes during long-term adaptation to artificially assisted environments, resulting in weakened resistance against powdery mildew. Conversely, wild honeysuckle populations rely on individual adaptability and genetic diversity within the population to thrive in complex natural environments while maintaining robust adaptability, including resilience against extreme temperatures and environmental stresses such as drought. Notably, they exhibit heightened resistance against biological stresses like pathogens. The enhancement of disease resistance has become an increasingly prominent focus in plant breeding programs worldwide. Wild plants possess significant breeding potential, including resistance to pathogenic microorganisms or the reintroduction of quality-related genes lost during prolonged breeding (Al-Ashkar et al., 2020), which can serve as a crucial complement to address the scarcity of existing crops while enhancing the innovation potential of crop varieties.

## 5 Conclusion

The results of this study showed that there were significant differences in botanical characters, phytochemical components and stress resistance between wild and cultivated varieties of honeysuckle. The cultivated honeysuckle exhibited a suite of distinctive morphological and biochemical attributes when compared to its wild progenitor. Notably, the cultivated variant displayed a more upright plant architecture, characterized by an increased plant height and a more erect growth habit. Furthermore, it possessed a higher flower-to-leaf ratio, indicative of a floral biomass emphasis, and elongated bud lengths. Biochemically, the cultivated honeysuckle demonstrated significantly enhanced concentrations of chlorogenic acid, a phenolic compound known for its various physiological roles. Despite these modifications, in the context of resistance to powdery mildew, the wild individuals exhibited a clear superiority over the cultivated strains, suggesting a trade-off between the desirable traits of cultivation and natural disease resistance. These differences have important guiding significance for the medicinal value and cultivation strategy of honeysuckle. The cultivated honeysuckle may have better efficacy due to its higher chemical content, while the wild honeysuckle may have unique advantages in terms of genetic diversity and stress resistance, so it is also worth further research and utilization.

## Data Availability

Data for this paper have been archived in figshare: https://figshare.com/articles/dataset/data_xlsx/28188950?file=51612644, further inquiries can be directed to the corresponding author.

## References

[B1] Abbo S. Lev-Yadun S. Gopher A. (2014). The 'Human Mind' as a common denominator in plant domestication. J. Exp. Bot. 65, 1917–1920. doi: 10.1093/jxb/eru068 24638899

[B2] Al-Ashkar I. Alderfasi A. Ben Romdhane W. Seleiman M. F. El-Said R. A. Al-Doss A. (2020). Morphological and genetic diversity within salt tolerance detection in eighteen wheat genotypes. Plants (Basel) 9. doi: 10.3390/plants9030287 PMC7154827 32106488

[B3] Choi J. H. Park Y. N. Li Y. Jin M. H. Lee J. Lee Y.. (2010). Flowers *of Inula japonica* Attenuate Inflammatory Responses. Immune Network 10, 145–152 . doi: 10.4110/in.2010.10.5.145 PMC2993946 21165243

[B4] Guo X. Y. Wang Y. W. Yu X. Yang R. Wang L. N. Zhang F.. (2022). Simultaneous determination of 11 active components in *Lonicera japonica* flowers and leaves at different development stages by HPLC-DAD. Zhongguo Zhong Yao Za Zhi 47, 2148–2157. doi: 10.19540/j.cnki.cjcmm.20220114.101 35531730

[B5] Guo Y. J. Luo T. Wu F. Mei Y. W. Peng J. Liu H.. (2015). Involvement of TLR2 and TLR9 in the anti-inflammatory effects of chlorogenic acid in HSV-1-infected microglia. Life Sci. 127, 12–18. doi: 10.1016/j.lfs.2015.01.036 25744394

[B6] Kim J.-Y. Lee Y.-S. Park E.-J. Lee H.-J. (2022). Honeysuckle berry (*Lonicera caerulea* L.) inhibits lipase activity and modulates the gut microbiota in high-fat diet-fed mice. Molecules 27. doi: 10.3390/molecules27154731 PMC9330072 35897908

[B7] Li L. Y. Wang Q. Deng L. Lin Z. Lin J. J. Wang X. Y.. (2023). Chlorogenic acid alleviates hypoxic-ischemic brain injury in neonatal mice. Neural Regener. Res. 18, 568–576. doi: 10.4103/1673-5374.350203 PMC9727453 36018179

[B8] Li M. Wang Y. Jin J. Dou J. Guo Q. Ke X.. (2020). Inhibitory activity of honeysuckle extracts against influenza A virus *in vitro* and *in vivo* . Virologica Sin. 36, 490–500. doi: 10.1007/s12250-020-00302-6 PMC8257810 33044658

[B9] Mboujda F. M. M. Avana-Tientcheu M.-L. Momo S. T. Ntongme A. M. Vaissayre V. Azandi L. N.. (2022). Domestication syndrome in dacryodes edulis (Burseraceae): comparison of morphological and biochemical traits between wild and cultivated populations. Plants 11. doi: 10.3390/plants11192496 PMC9571564 36235361

[B10] Meyer R. S. DuVal A. E. Jensen H. R. (2012). Patterns and processes in crop domestication: an historical review and quantitative analysis of 203 global food crops. New Phytol. 196, 29–48. doi: 10.1111/j.1469-8137.2012.04253.x 22889076

[B11] Muñoz N. Liu A. Kan L. Li M.-W. Lam H.-M. (2017). Potential uses of wild germplasms of grain legumes for crop improvement. Int. J. Mol. Sci. 18. doi: 10.3390/ijms18020328 PMC5343864 28165413

[B12] Parker I. M. López I. Petersen J. J. Anaya N. Cubilla-Rios L. Potter D. (2010). Domestication syndrome in caimito (Chrysophyllum cainito L.): fruit and seed characteristics. Econ Bot. 64, 161–175. doi: 10.1007/s12231-010-9121-4 20543881 PMC2882042

[B13] Pedrosa H. C. Clement C. R. Schietti J. (2018). The domestication of the amazon tree grape (*Pourouma cecropiifolia*) under an ecological lens. Front. Plant Sci. 9. doi: 10.3389/fpls.2018.00203 PMC5861524 29593750

[B14] Purugganan M. D. Fuller D. Q. (2009). The nature of selection during plant domestication. Nature 457, 843–848. doi: 10.1038/nature07895 19212403

[B15] Siqueira J. A. Batista-Silva W. Zsögön A. Fernie A. R. Araújo W. L. Nunes-Nesi A. (2023). Plant domestication: setting biological clocks. Trends Plant Sci. 28, 597–608. doi: 10.1016/j.tplants.2023.01.009 36822959

[B16] Vaughan D. A. Balazs E. Heslop-Harrison J. S. (2007). From crop domestication to super-domestication. Ann. Bot. 100, 893–901. doi: 10.1093/aob/mcm224 17940074 PMC2759215

[B17] Yang X. Yu A. Hu W. Zhang Z. Ruan Y. Kuang H.. (2023). Extraction, purification, structural characteristics, health benefits, and application of the polysaccharides from *lonicera japonica* thunb.: A review. Molecules 28. doi: 10.3390/molecules28124828 PMC10301136 37375383

